# Synthesis and Evaluation of Radiogallium-Labeled Peptide Probes for In Vivo Imaging of Legumain Activity

**DOI:** 10.3390/molecules30234527

**Published:** 2025-11-24

**Authors:** Takeshi Fuchigami, Kohnosuke Itagaki, Sakura Yoshida, Morio Nakayama, Masayuki Munekane, Kazuma Ogawa

**Affiliations:** 1 Laboratory of Clinical Analytical Sciences, Graduate School of Medical Sciences, Kanazawa University, Kakuma-machi, Kanazawa 920-1192, Ishikawa, Japan; munekane@p.kanazawa-u.ac.jp (M.M.); kogawa@p.kanazawa-u.ac.jp (K.O.); 2 Department of Hygienic Chemistry, Graduate School of Biomedical Sciences, Nagasaki University, 1-14 Bunkyo-machi, Nagasaki 852-8521, Nagasaki, Japannakayamam@nagasaki-u.ac.jp (M.N.); 3 Faculty of Environmental Engineering, The University of Kitakyushu, Kitakyushu 802-8577, Fukuoka, Japan; yoshida-s@kitakyu-u.ac.jp

**Keywords:** legumain, activatable cell penetrating peptide, radiogallium, cancer imaging, SPECT

## Abstract

Legumain (LGMN), a lysosomal cysteine protease, is crucial for tumor progression, invasion, and metastasis, making it a promising target for cancer imaging and therapy. This study developed novel ^67^Ga-labeled 1,4,7-triazacyclononane-1,4,7-triacetic acid (NOTA)-conjugated LGMN-cleavable peptide probes ([^67^Ga]Ga-NOTA-LCPs) composed of polyarginine and polyglutamic acid sequences linked by LGMN-cleavable sites for nuclear medicine imaging of LGMN activity. The probes were synthesized via fluorenylmethoxycarbonyl solid-phase peptide synthesis and radiolabeled in high radiochemical yields. In vitro assays with HCT116 cells showed significantly higher uptake of [^67^Ga]Ga-NOTA-LCPs compared to non-cleavable controls, confirming efficient cleavage and cellular uptake. In vivo studies in tumor-bearing mice revealed rapid renal clearance, low non-specific binding, and favorable tumor-to-blood ratios, particularly for [^67^Ga]Ga-NOTA-LCP-1. These results demonstrate the potential of [^67^Ga]Ga-NOTA-LCPs as effective LGMN-responsive imaging agents, with further optimization needed to improve tumor specificity and reduce off-target accumulation.

## 1. Introduction

Legumain (LGMN), also known as asparaginyl endopeptidase, is a lysosomal cysteine protease that plays crucial roles in various physiological and pathological processes, including extracellular matrix remodeling, exosome-mediated chemical transport, and angiogenesis within the tumor microenvironment [1,2]. LGMN overexpression has been reported in numerous solid tumors, including breast [3], colorectal [4], gastric, ovarian [5], and prostate [6] cancers, and it significantly contributes to tumor progression, invasion, and metastasis [7]. Elevated LGMN levels are frequently observed in tumor neovascular endothelial cells, tumor-associated macrophages, and the tumor stroma, highlighting its multifaceted role in the tumor microenvironment [4,6,8].

Mechanistically, LGMN has been implicated in the activation of matrix metalloproteinases (MMP-2, MMP-3, and MMP-9), which promote extracellular matrix degradation and facilitate cancer cell invasion and metastasis [1,9]. Furthermore, LGMN is associated with the activation of the PI3K/AKT signaling pathway [10], integrin-mediated adhesion [11,12], and transforming growth factor (TGF)-β1 signaling [13], all of which collectively enhance tumor growth, invasion, and metastasis. Notably, LGMN overexpression is correlated with aggressive tumor phenotypes and poor prognosis in colorectal and gastric cancers, whereas reduced LGMN expression has been linked to improved clinical outcomes [14,15]. Additionally, proteases play a role in disrupting oncogene regulation by interacting with p53 and other pathways, further contributing to tumorigenesis [16]. Given its prominent role in tumor biology, LGMN has been identified as a valuable biomarker for cancer diagnosis and prognosis as well as a promising therapeutic target. Its distinct expression profile and functional versatility make it an appealing candidate for developing diagnostic tools and therapeutic strategies to mitigate tumor progression and metastasis.

Therefore, LGMN-targeting molecules are expected to have a wide range of applications in elucidating cancer biological mechanisms in the tumor microenvironment, as well as in the diagnosis and treatment of tumors. In addition to its lysosomal localization, legumain has been reported to be partially expressed on the surface of tumor cells, where it can cleave extracellular substrates [17,18]. This aberrant membrane-associated activity provides a unique opportunity for selective molecular targeting in cancer. The surface-expressed form of legumain can thus serve as an accessible and tumor-specific biomarker, making it a promising target for the development of imaging probes and therapeutic agents aimed at tumor diagnosis and treatment. Nanoparticle drugs based on liposomes and nanogels that exhibit anticancer activity in response to LGMN have been developed with anticipated clinical applications [19,20,21]. Imaging agents for LGMN, such as fluorescent, magnetic resonance imaging (MRI), and ultrasound probes responsive to LGMN activity, have also been developed [22,23,24,25,26]. Furthermore, nuclear medicine imaging probes such as positron emission tomography (PET) and single-photon emission computed tomography (SPECT), which offer advantages such as superior detection depth and lower required dosages compared with the aforementioned imaging modalities, have also been reported [27,28]. LGMN-activatable self-assembly-type ^18^F-labeled PET probes demonstrate high tumor accumulation and retention, with the incorporation of a (His-Glu)_3_ linker improving the signal-to-noise ratio. However, non-specific liver uptake remains a concern [29,30]. Similarly, ^11^C-labeled PET tracers targeting LGMN exhibit high affinity. However, challenges with target specificity and metabolic stability still need to be addressed [31].

**Figure 1:**
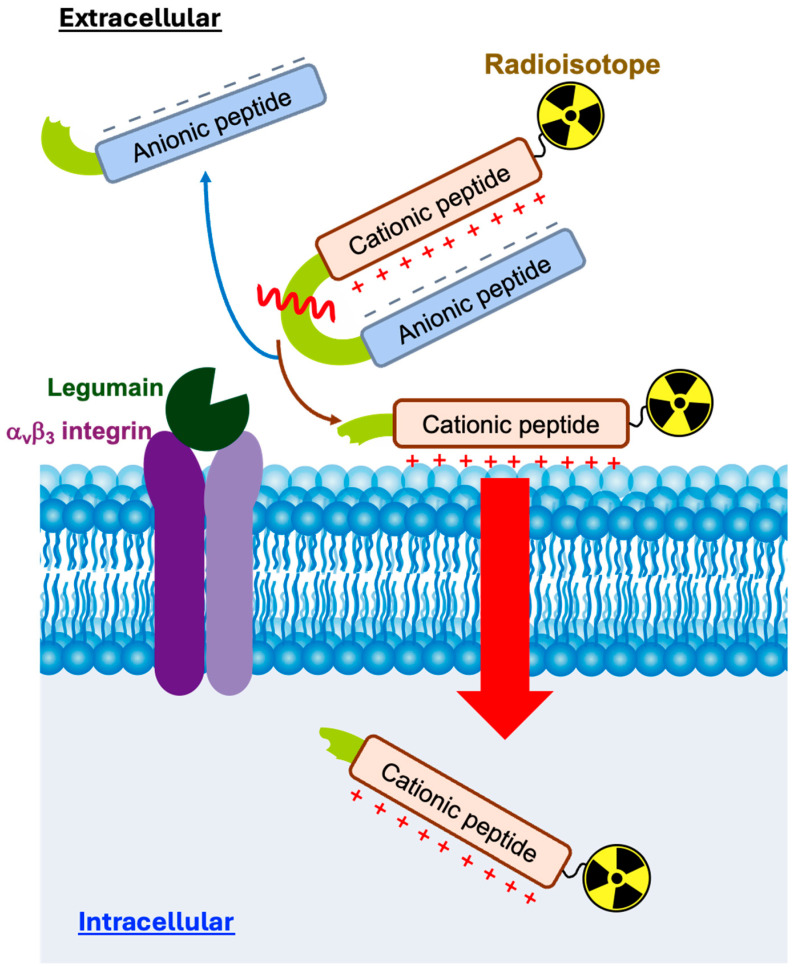
Strategy of peptide probes for nuclear medicine imaging of legumain activity in cancers. The cell-penetrating ability of a polycationic peptide is suppressed by coupling it with a polyanionic peptide. Upon cleavage of the linker (yellow-green) by legumain (LGMN), the polycationic peptide is released, facilitating the transport of attached radioisotopes across the cell membrane. The polyanionic peptide is eliminated from the target tissue.

We previously developed LGMN-activatable SPECT probes for noninvasive protease assessment, consisting of a radioiodinated d-Arg nonamer (d-Arg)_9_-based cell-penetrating peptide (CPP) and a negatively charged d-Glu nonamer (d-Glu)_9_ peptide linked by a LGMN-cleavable sequence [18]. These peptide probes function through enzyme-mediated cleavage of the linker, which disrupts the electrostatic interactions between cationic and anionic peptides [32,33,34]. Consequently, LGMN-cleavable probes were designed to minimize accumulation in cells lacking LGMN while enabling the cleaved cationic peptide to selectively penetrate and accumulate in the membranes of cancer cells with high LGMN expression (Figure 1). Jiang et al. reported that a fluorescent peptide containing an MMP-cleavable sequence inserted between (d-Arg)_9_ and (d-Glu)_9_ accumulated in tumor tissues of mice transplanted with human cancer cells, correlating with MMP expression [34]. Similarly, our previous study demonstrated significantly higher cellular uptake of an ^125^I-labeled peptide with an LGMN-cleavable sequence (Ala-Ala-Asn-Val, where the Asn-Val amide bond is cleavable [35,36]) between (d-Arg)_9_ and (d-Glu)_9_ ([^125^I]I-LCP) in HCT116 cells overexpressing LGMN, compared to its ^125^I-labeled LGMN-non-cleavable peptide (NCP) counterpart ([^125^I]I-NCP), which contains an Ala-Ala-Cit-Val sequence resistant to LGMN cleavage. However, in vivo tumor uptake of these peptides showed no significant differences in HCT116 tumor-bearing mice [18]. This suggests that not only the cleavage efficiency near tumor tissues but also the complex in vivo dynamics, such as accumulation in normal tissues and excretion pathways, play a role. The strong interaction between polyarginine and polyanions may result in suboptimal accumulation in target tissues and/or nonspecific accumulation in non-target tissues. Furthermore, in vivo tumor accumulation of LGMN-cleavable probe (LCP)-type peptides may be influenced by the labeling site. Therefore, modulating the binding strength between cationic and anionic peptides through electrostatic interactions, as well as altering the labeling site, could affect cellular uptake following cleavage by LGMN.

**Figure 2:**
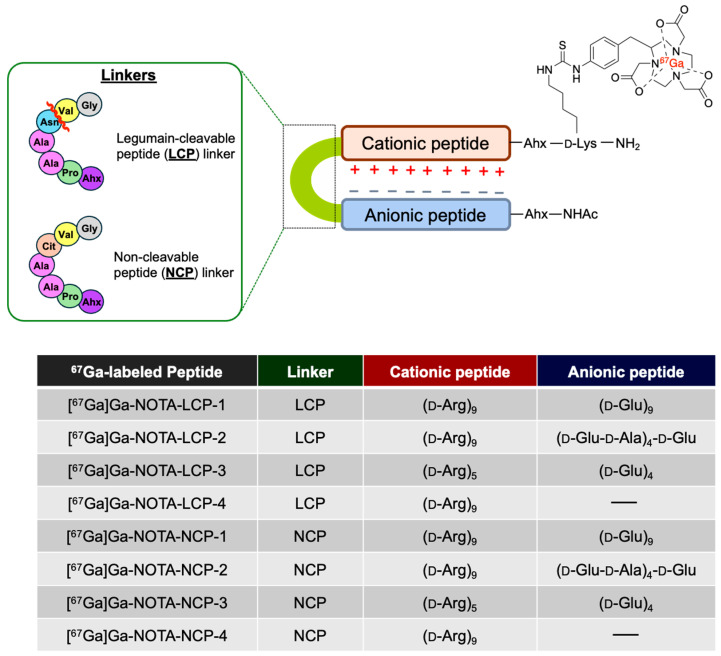
Designing the LGMN-cleavable peptides (LCP) involves fusing a cell membrane-permeable cationic peptide and an anionic peptide via a legumain-cleavable linker. Non-legumain-cleavable peptides (NCPs) have been designed as negative control peptides; asparagine residues in the cleavable linker have been displaced by citrulline. [^67^Ga]Ga-NOTA-LCP-4 and [^67^Ga]Ga-NOTA-NCP-4 (without (d-Glu)_9_) have been designed to examine the effect of anionic peptides. The legumain cleavage site is indicated by the jagged line. Ahx and Cit represent ε-aminohexanoic acid and citrulline, respectively.

In this study, we designed novel LCP probes by linking polyarginine-based cationic peptides ((d-Arg)_5_ or (d-Arg)_9_) with polyglutamic acid-based anionic peptides ((d-Glu)_4_, (d-Glu-d-Ala)_4_-d-Glu, or (d-Glu)_9_) via an LGMN-cleavable amino acid sequence (Figure 2). For the radiolabeling unit in nuclear medicine imaging, radiogallium-labeled 1,4,7-triazacyclononane-1,4,7-triacetic acid (NOTA) was selected because of its stable labeling properties. Although ^68^Ga, with its 68-min half-life, is a practical PET radionuclide that avoids the need for a cyclotron, we used ^67^Ga, a SPECT radionuclide with a longer 72 h half-life, as a more convenient alternative in this study. The fundamental properties of ^67^Ga-labeled LCPs were compared to those of corresponding ^67^Ga-labeled NCPs through in vitro cell assays and in vivo evaluations using tumor-bearing mice. This comparison provided valuable insights into the potential of ^67^Ga-labeled LCP as an effective in vivo imaging agent for LGMN activity.

## 2. Results and Discussion

### 2.1. Preparation of NOTA- and Ga-NOTA-Peptides

To evaluate how converting the radiolabeling site from *m*-[^125^I]iodobenzoyl ([^125^I]I-LCP) [18] to ^67^Ga-NOTA affects the properties of LGMN-targeting probes, we designed [^67^Ga]Ga-LCP-1 with an LGMN-cleavable sequence between (d-Arg)_9_ and (d-Glu)_9_. To enhance the efficient release of the cationic peptide after cleavage and its subsequent accumulation in tumor tissues by weakening the electrostatic interaction between cationic and anionic peptides, we designed [^67^Ga]Ga-NOTA-LCP-2 and [^67^Ga]Ga-NOTA-LCP-3. These probes incorporated (d-Arg)_9_ with (d-Glu-d-Ala)_4_-d-Glu or (d-Arg)_5_ with (d-Glu)_4_ as the cationic and anionic peptides, respectively. [^67^Ga]Ga-NOTA-LCP-4, containing only (d-Arg)_9_ as the cationic peptide, was designed to evaluate the effect of introducing an anionic peptide. To evaluate the potential of each peptide as an LGMN-responsive imaging probe, [^67^Ga]Ga-NOTA-NCP1 to [^67^Ga]Ga-NOTA-NCP4, in which the Asn essential for cleavage by LGMN was substituted with Cit, were also designed.

**Scheme 1:**
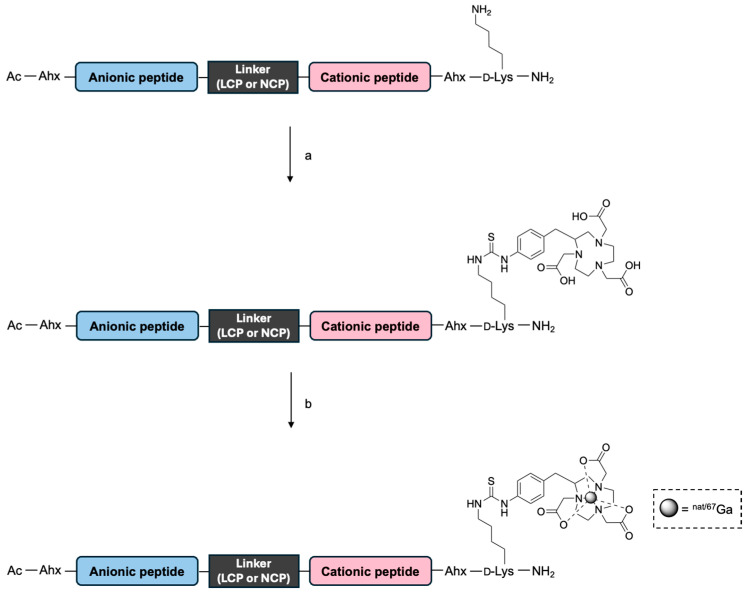
Synthesis of [^nat/67^Ga]Ga-NOTA-conjugated LCP or NCP derivatives. Reagents and conditions: (a) *p*-SCN-Bn-NOTA, 0.1 M sodium carbonate buffer (pH 9.5), r.t., 20 h; (b) Ga(NO_3_)_3_, 0.4 M acetate buffer (pH 4.0), 95 ˚C, 30 min (for [^nat^Ga]Ga-NOTA-peptides) or ^67^Ga-citrate, 0.6 M Phosphate buffer (pH 6.8), 95 ˚C, 30 min (for [^67^Ga]Ga-NOTA-peptides).

The LCP and NCP peptides were synthesized using standard fluorenylmethoxycarbonyl (Fmoc) solid-phase peptide synthesis. The cleaved peptides were conjugated with *p*-SCN-Bn-NOTA in 0.1 M carbonate buffer (pH 9.5), under which the ε-amino group of lysine is deprotonated and selectively reacts with the isothiocyanate moiety. the resulting NOTA-peptides, each containing a single NOTA conjugation, were obtained as NOTA-LCP-1, NOTA-LCP-2, NOTA-LCP-3, NOTA-LCP-4, NOTA-NCP-1, NOTA-NCP-2, NOTA-NCP-3, and NOTA-NCP-4 with yields of 65%, 19%, 32%, 50%, 31%, 18%, 13%, and 50%, respectively. Nonradioactive gallium chelation using Ga(NO_3_)_3_ resulted in the Ga-NOTA-peptides Ga-NOTA-LCP-1, Ga-NOTA-LCP-2, Ga-NOTA-LCP-3, Ga-NOTA-LCP-4, Ga-NOTA-NCP-1, Ga-NOTA-NCP-2, Ga-NOTA-NCP-3, and Ga-NOTA-NCP-4 with yields of 33%, 4%, 26% 50%, 19%, 9%, 17%, and 50%, respectively (Scheme 1). Target peptides were identified using MALDI-TOF MS (Table S1).

### 2.2. LGMN Cleavage Test of NOTA-Peptides

**Figure 3:**
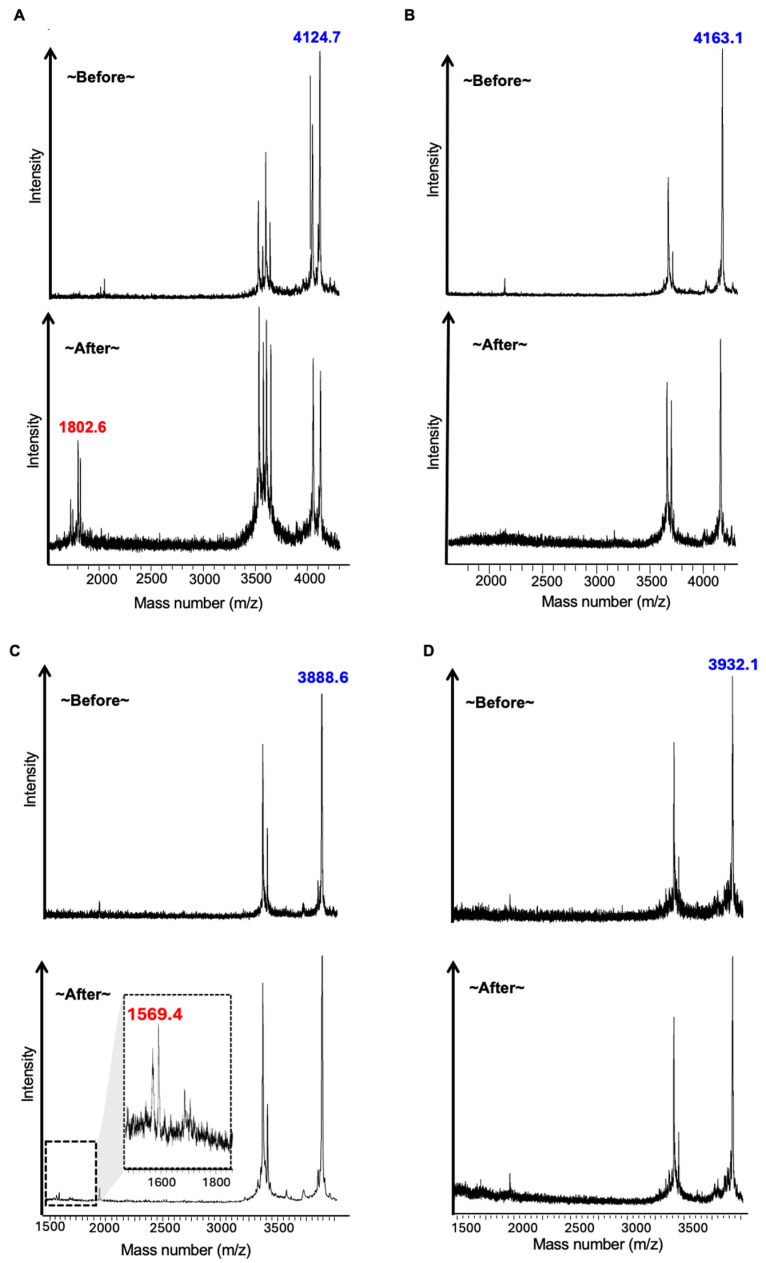

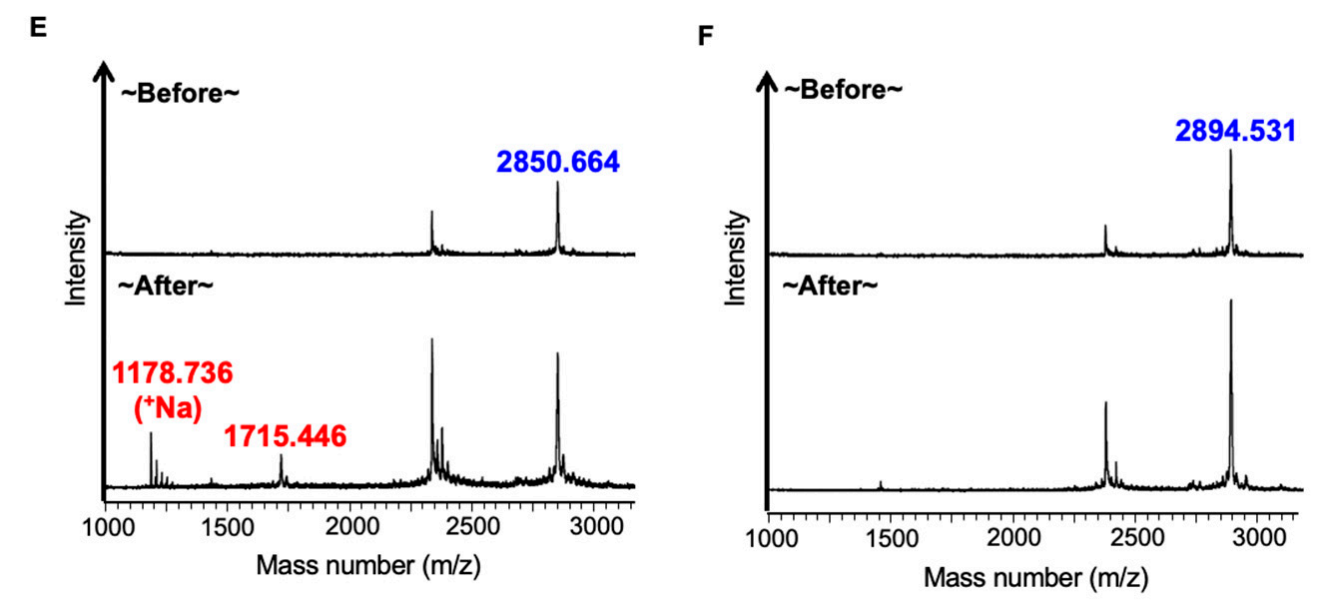
MALDI-TOF mass spectra of NOTA-LCP-1 (**A**), NOTA-NCP-1 (**B**), NOTA-LCP-2 (**C**), NOTA-NCP-2 (**D**), NOTA-LCP-3 (**E**), and NOTA-NCP-3 (**F**), before and after cleavage by recombinant human LGMN. The *m*/*z* values of the peptides are displayed in blue and red for pre- and post-cleavage, respectively.

To evaluate whether the NOTA-peptides were recognized as LGMN substrates, they were incubated with activated recombinant human LGMN (rhLGMN). MALDI-TOF-MS analysis before and after cleavage by rhLGMN revealed the generation of cleaved peptides with molecular weights of 1802.6 from NOTA-LCP-1, 1569.4 from NOTA-LCP-2, and 1178.7 and 1715 from NOTA-LCP-3 (Figure 3A, Figure 3C and Figure 3E, respectively). In contrast, all NOTA-NCP peptides remained uncleaved, and no significant cleavage products were detected after incubation with rhLGMN (Figure 3B, Figure 3D and Figure 3F, respectively). These results confirmed the suitability of NOTA-LCP1, NOTA-LCP2, and NOTA-LCP3 as scaffolds for LGMN imaging probes based on their ability to undergo LGMN-mediated cleavage.

### 2.3. Radiosynthesis of [^67^Ga]Ga-NOTA-Peptides

NOTA-LCP and NOTA-NCP peptides, precursors for ^67^Ga-radiolabeling, were reacted with [^67^Ga]Ga-citrate at 95 °C for 30 min to produce [^67^Ga]Ga-NOTA-LCP and [^67^Ga]Ga-NOTA-LCP peptides, respectively (Scheme 1). Following HPLC purification (Figures S1 and S2), the products [^67^Ga]Ga-NOTA-LCP-1, [^67^Ga]Ga-NOTA-LCP-2, [^67^Ga]Ga-NOTA-LCP-3, [^67^Ga]Ga-NOTA-LCP-4, [^67^Ga]Ga-NOTA-NCP-1, [^67^Ga]Ga-NOTA-NCP-2, [^67^Ga]Ga-NOTA-NCP-3, and [^67^Ga]Ga-NOTA-NCP-4 achieved radiochemical yields of 96%, 99%, 92%, 90%, 92%, 98%, 95%, and 90%, respectively. The radiosynthesis was performed using carrier-free ^67^Ga, resulting in a molar activity of 61.7 GBq/μmol for the [^67^Ga]Ga-NOTA-peptides. Since each precursor was separated from the product by HPLC, the purified [^67^Ga]Ga-NOTA-peptides are expected to retain essentially the same molar activity as the starting carrier-free ^67^Ga. The [^67^Ga]Ga-NOTA-peptides showed identical retention times to the corresponding non-radioactive Ga-NOTA-peptide standards and were therefore identified as the desired [^67^Ga]Ga-NOTA-peptides, with radiochemical purities exceeding 95% (Figures S3 and S4). The stability of [^67^Ga]Ga-NOTA-peptides was evaluated in 0.1 M phosphate buffer (pH 7.4) at 37 °C for 4 h, and more than 95% of the compounds remained unchanged.

### 2.4. In Vitro Cellular Uptake of [^67^Ga]Ga-NOTA-Peptides

**Figure 4:**
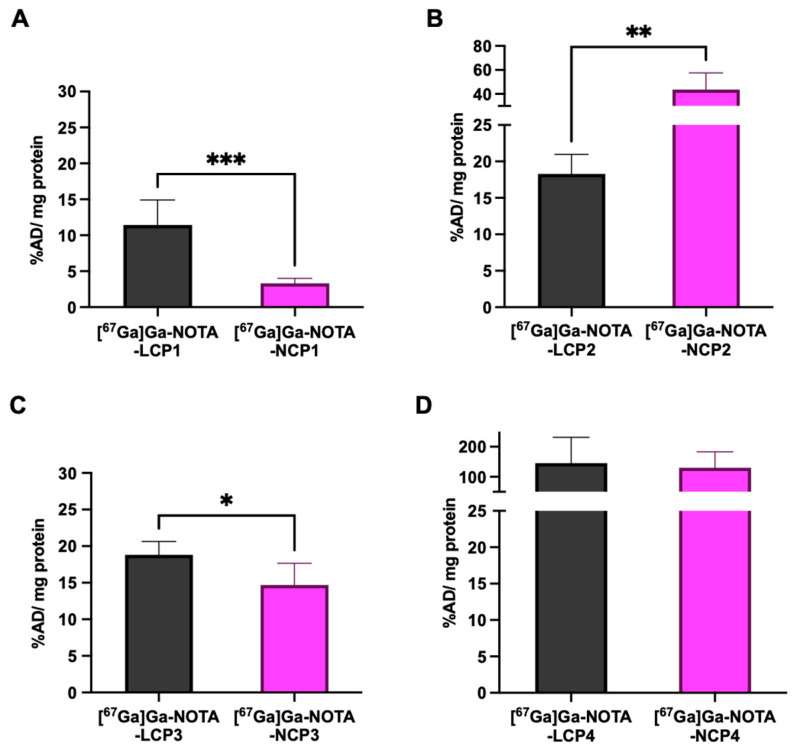
HCT116 cell uptake comparison between LCP and their NCP counterparts for (d-Arg)_9_(d-Glu)_9_ type (**A**), (d-Arg)_9_(d-Glu)_5_(d-Ala)_4_ type (**B**), (d-Arg)_5_(d-Glu)_4_ type (**C**), and (d-Arg)_9_ type (**D**). * *p* < 0.05, ** *p* < 0.01, *** *p* < 0.001 (unpaired *t*-test). Values are represented as the mean ± SD (*n* = 5–6).

The in vitro cellular uptake of [^67^Ga]Ga-NOTA-LCPs and [^67^Ga]Ga-NOTA-NCPs was evaluated using HCT116 cells, as shown in Figure 4. Similarly to [^125^I]I-LCP, which contains the same cationic and anionic peptides in previous studies [18], [^67^Ga]Ga-NOTA-LCP-1 exhibited significantly higher uptake compared to [^67^Ga]Ga-NOTA-NCP-1 (11.44 ± 3.46 and 3.32 ± 0.69 percent applied dose (AD) per milligram of protein (%AD/mg protein), respectively). Contrary to expectations, [^67^Ga]Ga-LCP-2 exhibited significantly lower cellular accumulation compared to [^67^Ga]Ga-NCP-2 (18.28 ± 2.68 and 43.61 ± 13.96 %AD/mg protein, respectively). It is hypothesized that the insertion of d-alanine between the d-glutamic acid residues within the anionic peptide weakens its electrostatic interactions with the cationic peptide, thereby promoting dissociation. However, this modification likely increases the hydrophobicity of the peptide, resulting in nonspecific binding to the cell membrane. [^67^Ga]Ga-NOTA-LCP-3 demonstrated significantly higher accumulation compared to [^67^Ga]Ga-NCP-3 (18.80 ± 1.84 and 14.69 ± 2.96 %AD/mg protein, respectively); however, the observed difference appeared smaller than that between [^67^Ga]Ga-NOTA-LCP-1 and [^67^Ga]Ga-NOTA-NCP-1. This outcome was hypothesized to result from the fewer residues in the cationic and anionic peptides of [^67^Ga]Ga-NOTA-LCP-3, which may have led to insufficient electrostatic interactions or inadequate cellular uptake of (d-Arg)_5_ following cleavage. Both [^67^Ga]Ga-NOTA-LCP-4 and [^67^Ga]Ga-NOTA-NCP-4, which lack anionic peptides, exhibited higher cellular accumulation compared to other peptides (145.20 ± 86.07 and 129.90 ± 53.60 %AD/mg protein, respectively). The much lower cellular accumulation of [^67^Ga]Ga-NOTA-LCP-1, which contains r9, compared to that of [^67^Ga]Ga-NOTA-LCP-4, suggests that a portion of the peptide may not dissociate even after enzymatic cleavage. Notably, while [^67^Ga]Ga-NOTA-LCP2 and LCP4 showed higher cellular uptake than [^67^Ga]Ga-LCP1 and [^67^Ga]Ga-LCP3, the corresponding [^67^Ga]Ga-NOTA-[^67^Ga]Ga-NCP2 and [^67^Ga]Ga-NCP4 also exhibited similarly high uptake, suggesting that this accumulation is largely non-specific. In contrast, [^67^Ga]Ga-NOTA-LCP1 and [^67^Ga]Ga-LCP3 showed higher uptake than their NCP counterparts ([^67^Ga]Ga-NCP1 and [^67^Ga]Ga-NCP3), indicating enzyme-dependent specific accumulation. Therefore, [^67^Ga]Ga-LCP1 and [^67^Ga]Ga-LCP3 were selected for subsequent in vivo evaluation.

### 2.5. In Vivo Biodistribution of [^67^Ga]Ga-NOTA-Peptides

**Figure 5:**
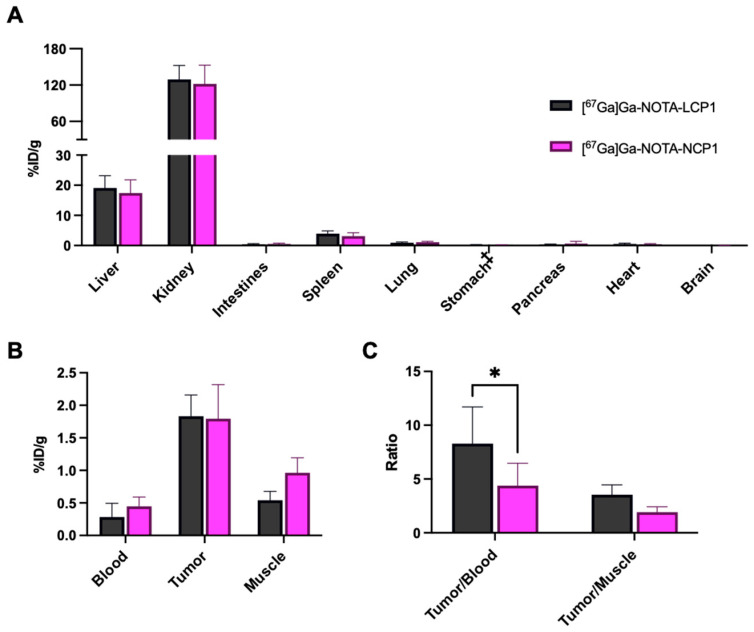
In vivo biodistribution of [^67^Ga]Ga-NOTA-LCP1 and [^67^Ga]Ga-NOTA-NCP1 in peripheral organs (**A**), blood, tumor, and muscle (**B**), and tumor-to-blood and tumor-to-muscle ratios (**C**) in HCT116 tumor-bearing mice at 1 h post-intravenous injection. Data are expressed as the percent injected dose per gram of tissue (%ID/g). * *p* < 0.05 (unpaired *t*-test). Values are presented as the mean ± SD (*n* = 5). ^‡^ Expressed as %ID.

**Figure 6:**
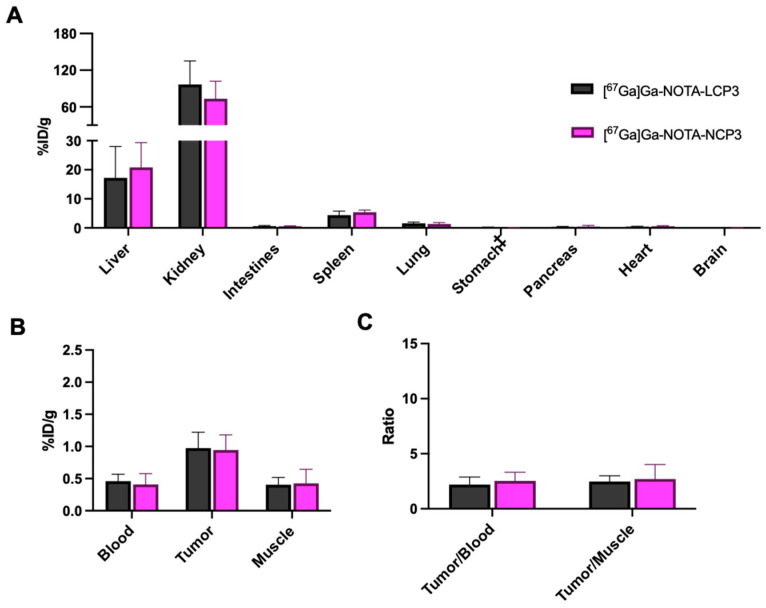
In vivo biodistribution of [^67^Ga]Ga-NOTA-LCP3 and [^67^Ga]Ga-NOTA-NCP3 in peripheral organs (**A**), blood, tumor, and muscle (**B**), and tumor-to-blood and tumor-to-muscle ratios (**C**) in HCT116 tumor-bearing mice at 1 h post-intravenous injection. Data are expressed as the %ID/g. Values are presented as the mean ± SD (*n* = 5). ^‡^ Expressed as %ID.

[^67^Ga]Ga-NOTA-LCP-1 and [^67^Ga]Ga-NOTA-LCP-3 demonstrated significantly higher cellular accumulation than [^67^Ga]Ga-NOTA-NCP-1 and [^67^Ga]Ga-NOTA-NCP-3, respectively. Consequently, we conducted in vivo biodistribution studies of these two [^67^Ga]Ga-NOTA-LCPs in HCT116 tumor-bearing mice and compared them with their corresponding [^67^Ga]Ga-NOTA-NCPs (Figure 5 and Figure 6). The detailed accumulation data are presented in Table S2. For all ^67^Ga-NOTA-peptides, the blood radioactivity concentration was low 1 h after administration in mice, with notable accumulation observed in both the liver and kidneys, the latter showing higher uptake. This suggests that there was minimal nonspecific binding in the bloodstream and the excretion pathway can be mainly through kidneys. The relatively high accumulation in the liver and kidneys may be partly explained by different mechanisms. For the liver, previous studies have reported that polyarginine-containing peptides exhibit high uptake in Kupffer cells, which could contribute to the observed hepatic accumulation [37]. In the kidneys, the peptides may be filtered through the renal glomeruli and subsequently degraded within kidney tissue, resulting in retention within renal cells. While legumain is expressed at low levels in the liver and moderate levels in the kidney [38], the observed biodistribution appears to result primarily from the physicochemical properties of the peptide, such as charge interactions. No significant differences in accumulation across various organs, including tumor tissues, were observed between [^67^Ga]Ga-NOTA-LCP-1 and [^67^Ga]Ga-NOTA-NCP-1 (1.83 ± 0.32 and 1.80 ± 0.51 %ID/g, respectively). However, [^67^Ga]Ga-NOTA-LCP-1 exhibited a significantly higher tumor-to-blood ratio than [^67^Ga]Ga-NOTA-NCP-1 (8.29 ± 3.41 and 4.39 ± 2.07, respectively). This suggests that [^67^Ga]Ga-NOTA-LCP1 may have been cleaved near the tumor tissue, enabling it to accumulate more efficiently in the tumor than [^67^Ga]Ga-NOTA-NCP-1. Conversely, the incorporation of l-Cit, a non-natural amino acid, into the linker region may have increased its resistance to enzymatic degradation relative to that of [^67^Ga]Ga-NOTA-NCP-1, which is composed entirely of natural amino acids, potentially influencing its blood retention properties. [^67^Ga]Ga-NOTA-LCP-3 demonstrated lower tumor accumulation (0.97 ± 0.25 %ID/g) and a reduced tumor-to-blood ratio (2.20 ± 0.69) compared to [^67^Ga]Ga-NOTA-NCP-1. Although this result contrasts with the trend observed in in vitro cellular uptake, it likely stems from factors such as the dissociation of cationic and anionic peptides in vivo and other pharmacokinetic influences. In contrast to [^67^Ga]Ga-NOTA-LCP-1 and [^67^Ga]Ga-NOTA-NCP-1, no significant differences in accumulation across any organ, including tumor tissue and tumor-to-blood ratio, were observed between [^67^Ga]Ga-NOTA-LCP-3 and [^67^Ga]Ga-NOTA-NCP-3. It remains uncertain whether this is attributable to the low efficiency of (d-Arg)_5_ insertion into tumor cells or to the minimal differences in blood stability and retention between these radioligands. Another possibility is the excess d-Arg residue in [^67^Ga]Ga-NOTA-NCP-3, which might enhance cellular uptake and thereby reduce the contrast with [^67^Ga]Ga-NOTA-LCP-3. Previous studies have demonstrated successful in vivo imaging of tumors using (d-Arg)_5_(d-Glu)_4_-type photoacoustic probes [39,40]. In contrast, the nuclear imaging agents in the present study showed limited differentiation in tumor uptake in vivo. Differences in the target enzyme and its recognition sequence may also have contributed to these findings. Nevertheless, these findings indicate that the length of the cationic peptide sequence is a critical factor for improving tumor accumulation.

This study provides important insights into the development of LGMN-selective activity-responsive imaging probes based on in vitro and in vivo studies. The analyses demonstrated that factors such as electrostatic interactions between cationic and anionic peptides, peptide lipophilicity, and charge significantly influenced probe performance, highlighting the need to reduce renal accumulation as a critical consideration. Building upon [^67^Ga]Ga-NOTA-LCP-1 as the lead compound, future development could focus on enhancing blood retention and tumor tissue accessibility, paving the way for its application as an effective LGMN imaging probe. For instance, we have previously improved blood retention and tumor accumulation of ^211^At-labeled RGD peptides by incorporating an albumin binding moiety [41]. Similarly, further structural optimization of LGMN-targeted peptide probes could enhance their specificity and functionality, establishing them as nuclear medicine imaging agents capable of selectively visualizing LGMN activity. These advancements hold promise for advancing cancer diagnosis and treatment through precise and targeted imaging strategies.

## 3. Materials and Methods

### 3.1. General Information

All reagents were purchased from commercial suppliers and utilized as received without additional purification unless explicitly mentioned. Fmoc-ε-aminohexanoic acid (Ahx-OH) was sourced from Merck Millipore (Bedford, MA, USA), whereas other Fmoc amino acids, along with 2-(1H-benzotriazole-1-yl)-1,1,3,3-tetramethyluronium hexafluorophosphate (HBTU), hydroxybenzotriazole (HOBt), and Fmoc-NH SAL Resin, were obtained from Watanabe Chemical (Hiroshima, Japan). [^67^Ga]Ga-citrate was purchased from PDR Pharma (Tokyo, Japan). Mass spectrometry (MS) data were acquired using matrix-assisted laser desorption/ionization time-of-flight (MALDI-TOF MS)-MS, either with an Ultraflex MALDI-TOF/TOF-MS system from Bruker Daltonics (Bremen, Germany) or an AXIMA Resonance system from Shimadzu (Kyoto, Japan). High-performance liquid chromatography (HPLC) was performed using a Shimadzu HPLC system consisting of an LC-10AT pump and SPD-10A UV detector set at 254 nm. A gamma survey meter (Aloka, Tokyo, Japan) was used as an RI detector. Radioactivity was measured using a 2470 WIZARD2 automated gamma counter equipped with a NaI (Tl) detector (PerkinElmer Life Sciences (Boston, MA, USA). The detection limit of the gamma counter used in this study is estimated to be approximately 100 cpm, and its linear range extends from 100 to 1,000,000 cpm. All samples measured in both the in vitro and in vivo experiments were within this linear range.

### 3.2. Solid-Phase Peptide Synthesis

Peptide synthesis was performed using the N-9-Fmoc stepwise solid-phase method, employing Fmoc amino acids in 100–150 mg of Fmoc-NH-SAL resin (0.55 mmol amine/g resin). The resin was soaked overnight, and the Fmoc group was removed using 20% (*v*/*v*) piperidine in *N*,*N*-dimethylformamide (DMF). After thorough washing to remove the piperidine, Fmoc amino acid (3 equiv), HBTU/HOBt (3 equiv), and *N*,*N*-diisopropylethylamine (DIPEA; 6 equiv) dissolved in 1.2 mL of DMF were added to initiate the coupling reaction. Each elongated peptide was cleaved from the resin by treatment with a mixture of trifluoroacetic acid (TFA), H_2_O, triisopropylsilane (TIS), and 1,2-ethanedithiol (EDT) (94:2.5:1:2.5, *v*/*v*/*v*/*v*) for 90 min under gentle agitation at room temperature. The resulting filtrate was precipitated using diethyl ether chilled to 4 °C, then centrifuged at 2500 rcf for 5 min. The precipitate was washed three times with diethyl ether and centrifuged between each washing step. Crude products were purified via a reverse-phase (RP) HPLC using Cosmosil C18 columns (Nacalai Tesque; 5C18-AR-II, 10 × 250 mm, Kyoto, Japan) with a water-acetonitrile gradient containing 0.1% TFA at a flow rate of 2.0 mL/min. The purified peptides were characterized using MALDI-TOF-MS.

### 3.3. Synthesis of NOTA-Peptides

Each peptide synthesized via solid-phase synthesis was dissolved in 0.1 M sodium carbonate buffer (pH 9.5) (500 µL, 2 mM) and reacted with 2-*S*-(4-isothiocyanatobenzyl)-1,4,7-triazacyclononane-1,4,7-triacetic acid (*p*-SCN-Bn-NOTA, 1.12 mg, 2.0 µmol) at room temperature for 20 h. The products were purified by RP-HPLC using Cosmosil C18 columns (Nacalai Tesque; 5C18-AR-II, 10 × 250 mm) with a gradient of 0.1% TFA in H_2_O and 0.1% TFA in CH_3_CN (from 90:10 to 60:40) at a flow rate of 2.0 mL/min. The identities of the purified NOTA-peptides were confirmed by MALDI-TOF MS. The purified NOTA-peptides were lyophilized and stored as aliquots. Before each experiment, the peptides were freshly dissolved in the appropriate buffer for radiosynthesis or biological assays, depending on the experimental conditions.

### 3.4. Synthesis of Ga-NOTA-Peptides

Ga-NOTA-peptides were synthesized as previously reported, with minor modifications [42]. For the synthesis of Ga-NOTA-peptides, each NOTA-peptide (0.3–0.5 μmol) and Ga(NO_3_)_3_·xH_2_O (0.09–0.13 mg) was dissolved in 800 μL of 0.4 M acetate buffer (pH 4.0), and heated at 95 °C for 30 min. Subsequently, the solution was purified by RP-HPLC using Cosmosil C18 columns (Nacalai Tesque; 5C18-AR-II, 10 × 250 mm) with a gradient of 0.1% TFA in H_2_O and 0.1% TFA CH_3_CN (from 90:10 to 60:40). The identities of the purified Ga-NOTA-peptides were confirmed by MALDI-TOF MS.

### 3.5. Synthesis of [^67^Ga]Ga-NOTA Peptides

The solution of NOTA-peptides (25 µL, 50 nmol) in 0.6 M phosphate buffer (pH 6.8, 2 mM) were reacted with 50 µL (0.1 mCi) of [^67^Ga]Ga-gallium citrate (3.7 MBq) at 95 °C for 30 min. The radiolabeled peptide was purified by reverse-phase HPLC using (Nacalai Tesque; 5C18-AR-II, 4.6 × 150 mm) a gradient of 0.1% TFA in H_2_O and 0.1% TFA in CH_3_CN, and the product was identified by matching its HPLC absorbance at 220 nm with that of a non-radioactive standard peptide. After purification, the solvent was removed using an evaporator, and the residues were dissolved in medium for cell uptake studies or in saline for in vivo studies.

### 3.6. LGMN Cleavage Assay

LGMN cleavage assays were performed as previously described [11]. In brief, 0.1 mg/mL of recombinant human LGMN protein (2199-CY-010, R&D Systems, Minneapolis, MN, USA) was activated by incubating pro-LGMN at pH 4.0 in a buffer containing 50 mM CH_3_CO_2_Na and 100 mM NaCl at 37 °C for 2 h. Peptides (0.5 mM) in 2-(*N*-morpholino)ethanesulfonic acid (MES) buffer (pH 5.0) were then reacted with activated LGMN (0.5 mg/mL) at 37 °C for 18 h. The reaction mixture was treated with 1.0 M NaOH, centrifuged at 9100 rcf for 1 min at 4 °C, and the supernatant was analyzed using MALDI-TOF MS.

### 3.7. Cellular Uptake Study

HCT116 cells were obtained from RIKEN BRC (Tsukuba, Japan). HCT116 cells were cultured to confluence in 12-well plates and rinsed with phosphate-buffered saline (PBS). [^67^Ga]Ga-NOTA-peptides (1.0 kBq, 0.6 mL) prepared in growth medium were added to each well and incubated at 37 °C for 2 h. Following incubation, the cells were washed twice with 0.5 mL of PBS containing 20 units of heparin, then lysed with cell lysis buffer M (Fujifilm Wako, Osaka, Japan) at 37 °C for 20 min. The radioactivity of the lysates was quantified using a gamma counter, and the protein concentration was measured using a Bradford protein assay kit (Bio-Rad, Hercules, CA, USA). The cellular uptake was expressed as the %AD/mg protein.

### 3.8. Tumor Xenograft Model

Animal experiments were conducted in accordance with our institutional guidelines and were approved by the Nagasaki University Animal Care Committee and the Kanazawa University Animal Care Committee. The animal experimental protocols were approved by the Committee on Animal Experimentation of Nagasaki University and Kanazawa University. BALB/c nu/nu mice (female; 4 weeks old) were supplied by Japan SLC (Shizuoka, Japan). The mice were maintained in a room at a constant ambient temperature and a 12/12 h light/dark cycle and were given free access to food and water. Mice were subcutaneously injected on their right shoulders with approximately 1.0 × 10^7^ HCT116 cells. The tumors were allowed to reach 300–500 mm^3^ (1–2 weeks after inoculation) before biodistribution studies were performed.

### 3.9. Biodistribution of [^67^Ga]Ga-NOTA-Peptides in Tumor-Bearing Mice

Each [^67^Ga]Ga-NOTA-peptide (7.4 kBq) was administered to mice via tail vein injection in 100 μL of saline. The mice were euthanized 1 h after injection, and their organs were harvested. The tissues were weighed and the radioactivity in each sample was measured using an automated gamma counter (*n* = 5). The results are expressed as the percent injected dose per gram of tissue (%ID/g) or as %ID.

### 3.10. Statistical Analysis

Differences in the cellular uptake of [^67^Ga]Ga-NOTA-peptides were analyzed using an unpaired *t*-test (Figure 4). Differences in the biodistribution of [^67^Ga]Ga-NOTA-peptides in mice were assessed using an unpaired *t*-test (Figure 5 and Figure 6). A *p*-value of <0.05 was considered statistically significant.

## 4. Conclusions

We designed and synthesized LGMN activity-responsive peptide probes that demonstrated selective enzymatic cleavage and sequence-specific accumulation in LGMN-overexpressing cancer cells. [^67^Ga]Ga-NOTA-LCP-1 showed a significantly higher tumor-to-blood ratio than [^67^Ga]Ga-NOTA-NCP-1, whereas nonspecific accumulation in other peptides was influenced by charge interactions and pharmacokinetics. Optimizing the charge design and labeling sites is expected to improve diagnostic accuracy and therapeutic potential. This study underscores the potential of non-invasive imaging of LGMN activity for advancing cancer diagnosis and treatment.

## Data Availability

The original contributions presented in the study are included in the article/Supplementary Materials, further inquiries can be directed to the corresponding author.

## Supplementary Materials

The following supporting information can be downloaded at: https://www.mdpi.com/article/10.3390/molecules30234527/s1, Table S1: MALDI-TOF-MS data of NOTA-peptides and Ga-NOTA-peptides; Figure S1: HPLC chromatograms of [^67^Ga]Ga-NOTA-LCPs during purification; Figure S2: HPLC chromatograms of [^67^Ga]Ga-NOTA-NCPs during purification; Figure S3: HPLC chromatograms of purified [^67^Ga]Ga-NOTA-LCPs; Figure S4: HPLC chromatograms of purified [^67^Ga]Ga-NOTA-NCPs; Table S2: Biodistribution of radioactivity of [^67^Ga]Ga-NOTA-peptides.

## Abbreviations

The following abbreviations are used in this manuscript:

Ahx-OH	Aminohexanoic acid
CPP	cell-penetrating peptide
DIPEA	*N*,*N*-Diisopropylethylamine
DMF	*N*,*N*-Dimethylformamide
EDT	1,2-Ethanedithiol
Fmoc	Fluorenylmethoxycarbonyl
HBTU	2-(1H-benzotriazole-1-yl)-1,1,3,3-tetramethyluronium hexafluorophosphate
HOBt	Hydroxybenzotriazole
RT-HPLC	Reverse-phase-high-performance liquid chromatography
LCP	LGMN-cleavable peptide
LGMN	Legumain
MALDI-TOF	Matrix-assisted laser desorption/ionization time-of-flight
MES	2-(*N*-Morpholino)ethanesulfonic acid
MMP	Matrix metalloproteinases
MRI	Magnetic resonance imaging
MS	Mass spectrometry
NCP	Non-cleavable peptide
NOTA	1,4,7-Triazacyclononane-1,4,7-triacetic acid
PBS	Phosphate-buffered saline
PET	Positron emission tomography
SPECT	Single-photon emission computed tomography
TFA	Trifluoroacetic acid
TGF	Transforming growth factor
TIS	Triisopropylsilane
