# Supplementary material for: Synthesis and Evaluation of Radiogallium-Labeled Peptide Probes for In Vivo Imaging of Legumain Activity
Source: Molecules. 2025 Nov 24;30(23):4527. doi: 10.3390/molecules30234527 (PMC12693633; doi:10.3390/molecules30234527)
Supplement: Supplementary file 1 [file molecules-30-04527-s001.zip › molecules-4013427-supplementary.pdf]

Supporting Information

Synthesis and evaluation of radiogallium-labeled peptide probes for in vivo imaging of legumain activity

Takeshi Fuchigami[1]*, Kohnosuke Itagaki[2], Sakura Yoshida[3], Morio Nakayama[2], Masayuki Munekane[1], Kazuma Ogawa[1]

[1] Laboratory of Clinical Analytical Sciences, Graduate School of Medical Sciences, Kanazawa University, Kakuma-machi, Kanazawa, Ishikawa 920-1192, Japan; t-fuchi@p.kanazawa-u.ac.jp (T. F.); munekane@p.kanazawa-u.ac.jp (M. M.); kogawa@p.kanazawa-u.ac.jp (K. O.)

[2] Department of Hygienic Chemistry, Graduate School of Biomedical Sciences, Nagasaki University, 1-14 Bunkyo-machi, Nagasaki 852-8521, Japan; mmtpc552@gmail.com (K.I.); nakayamam@nagasaki-u.ac.jp (M.N.)

[3] Faculty of Environment Engineering, The University of Kitakyushu, Kitakyushu, Fukuoka, Japan; yoshida-s@kitakyu-u.ac.jp (S.Y.)

* Correspondence: t-fuchi@p.kanazawa-u.ac.jp; Tel.: +81-76-234-4460 (T.F.)

Table of Contents

Table S1 MALDI-TOF-MS data of NOTA-peptides and Ga-NOTA-peptides ······· S3

Figure S1 HPLC chromatograms of [^{67}Ga]Ga-NOTA-LCPs during purification ···· S4

Figure S2 HPLC chromatograms of [^{67}Ga]Ga-NOTA-NCPs during purification ···· S5

Figure S3 HPLC chromatograms of purified [^{67}Ga]Ga-NOTA-LCPs ················ S6

Figure S4 HPLC chromatograms of purified [^{67}Ga]Ga-NOTA-NCPs ················ S7

Table S2 Biodistribution of radioactivity of [^{67}Ga]Ga-NOTA peptides ·············· S8

Table S1. MALDI-TOF-MS data of NOTA-peptides and Ga-NOTA-peptides.

Peptides	Calculated MS $(M + H)^+$	Observed MS $(M + H)^+$
NOTA-peptides		
NOTA-LCP1	4055.4	4054.4
NOTA-LCP2	3823.2	3824.2
NOTA-LCP3	2785.1	2784.1
NOTA-LCP4	2779.2	2781.3
NOTA-NCP1	4098.4	4098.2
NOTA-NCP2	3866.3	3865.3
NOTA-NCP3	2828.1	2828.0
NOTA-NCP4	2822.3	2822.2
Ga-*NOTA-peptides*		
Ga-NOTA-LCP1	4125.1	4121.0
Ga-NOTA-LCP2	3892.9	3890.9
Ga-NOTA-LCP3	2854.8	2851.2
Ga-NOTA-LCP4	2848.9	2848.4
Ga-NOTA-NCP1	4168.1	4163.5
Ga-NOTA-NCP2	3936.0	3932.5
Ga-NOTA-NCP3	2897.9	2894.4
Ga-NOTA-NCP4	2892.0	2892.0

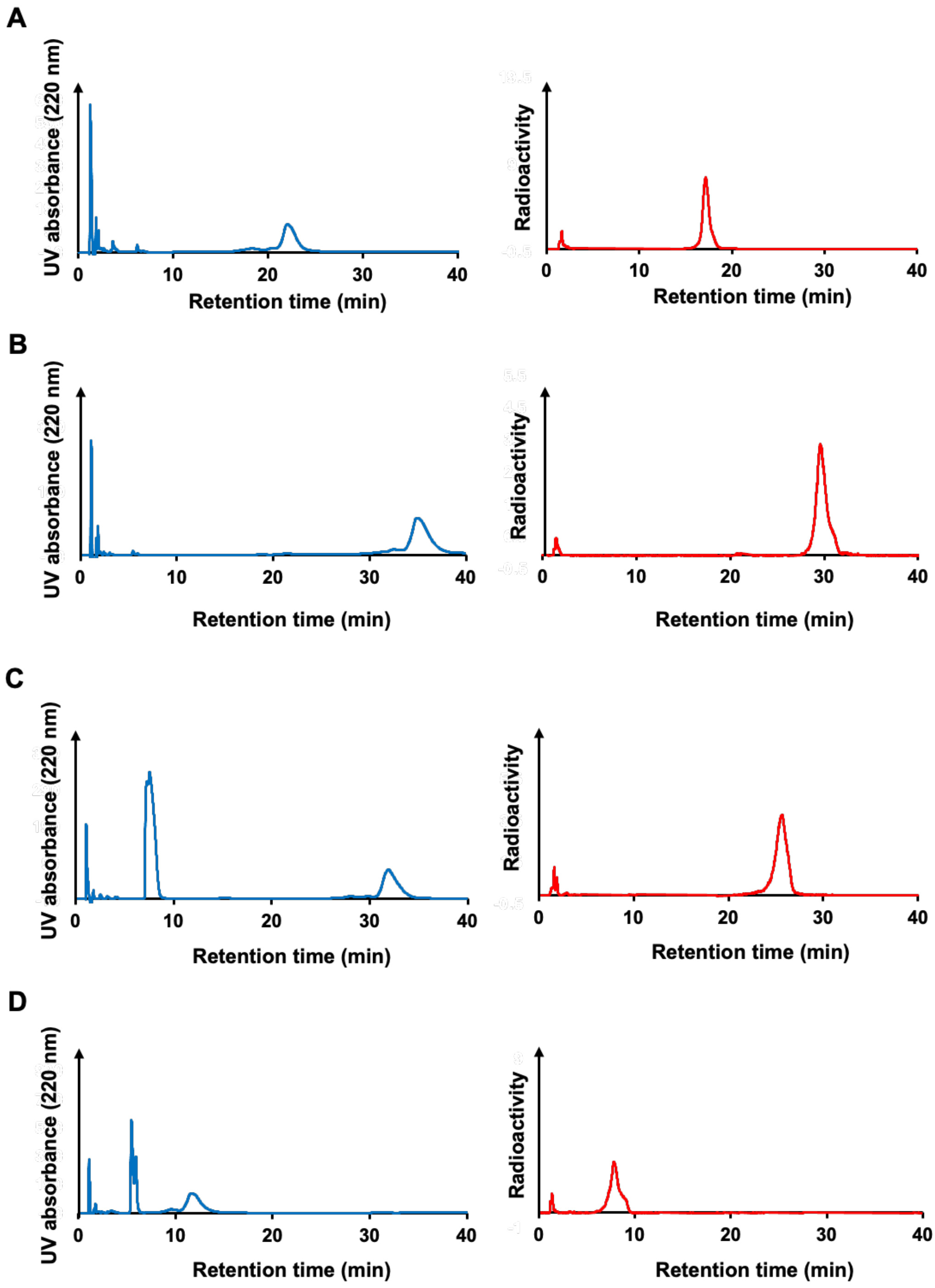

Figure S1. HPLC chromatograms of crude [^{67}Ga]Ga-NOTA-LCP1 (A), [^{67}Ga]Ga-NOTA-LCP2 (B), [^{67}Ga]Ga-NOTA-LCP3 (C), and [^{67}Ga]Ga-NOTA-LCP4 (D) during purification using gradient elution of 0.1% TFA in H_2O and 0.1% TFA in CH_3CN for 40 min. The gradient conditions were 84/16–78/22 (A), 84/16–78/20 (B), 87/13–81/19 (C), 84/16–80/20 (D), respectively, with a flow rate of 1.5 mL/min.

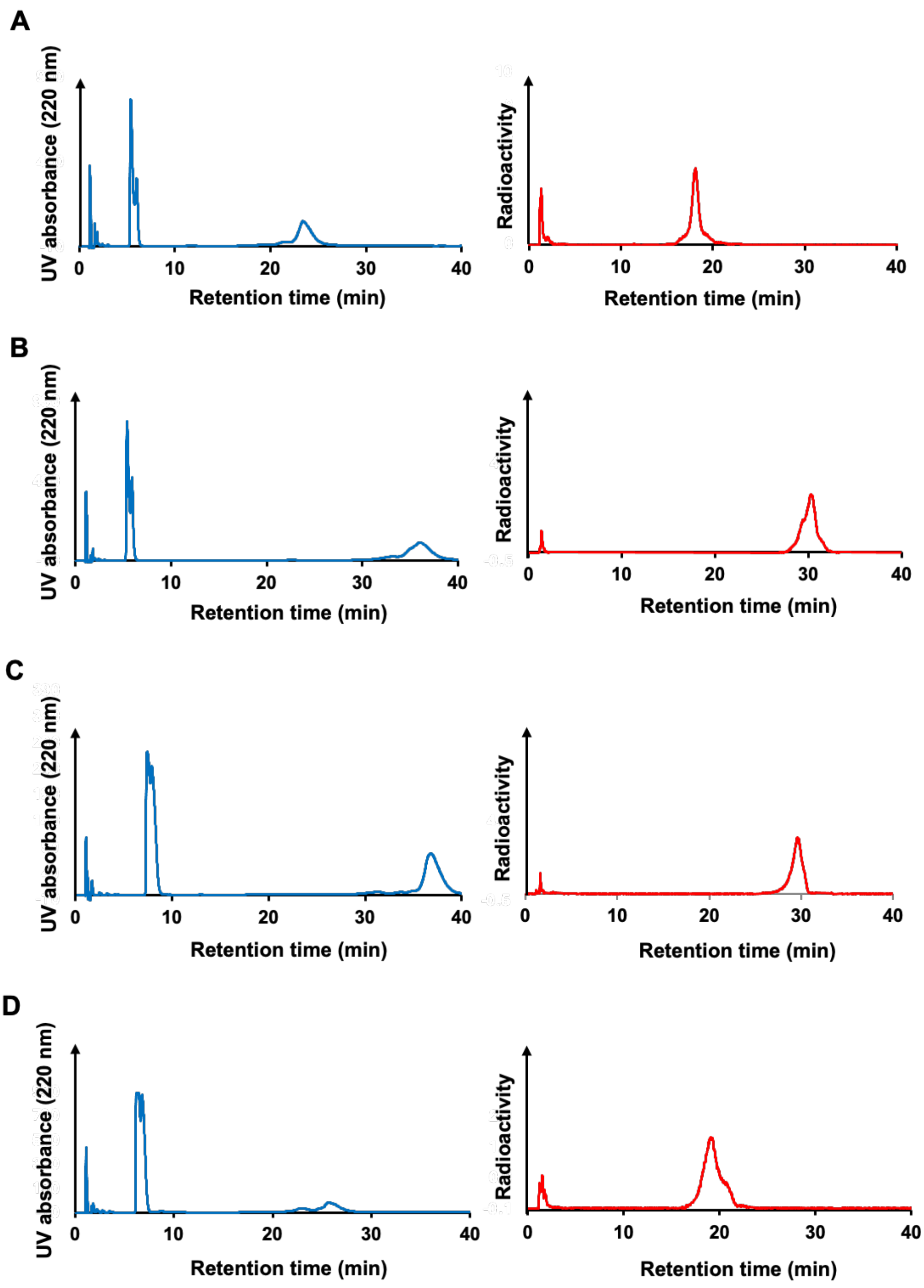

Figure S2. HPLC chromatograms of crude [^{67}Ga]Ga-NOTA-NCP1 (A), [^{67}Ga]Ga-NOTA-NCP2 (B), [^{67}Ga]Ga-NOTA-NCP3 (C), and [^{67}Ga]Ga-NOTA-NCP4 (D) during purification using gradient elution of 0.1% TFA in H_2O and 0.1% TFA in CH_3CN for 40 min. The gradient conditions were 84/16–78/21 (A), 84/16–78/20 (B), 87/13–81/19 (C), and 86/14–81/19 (D), respectively, with a flow rate of 1.5 mL/min.

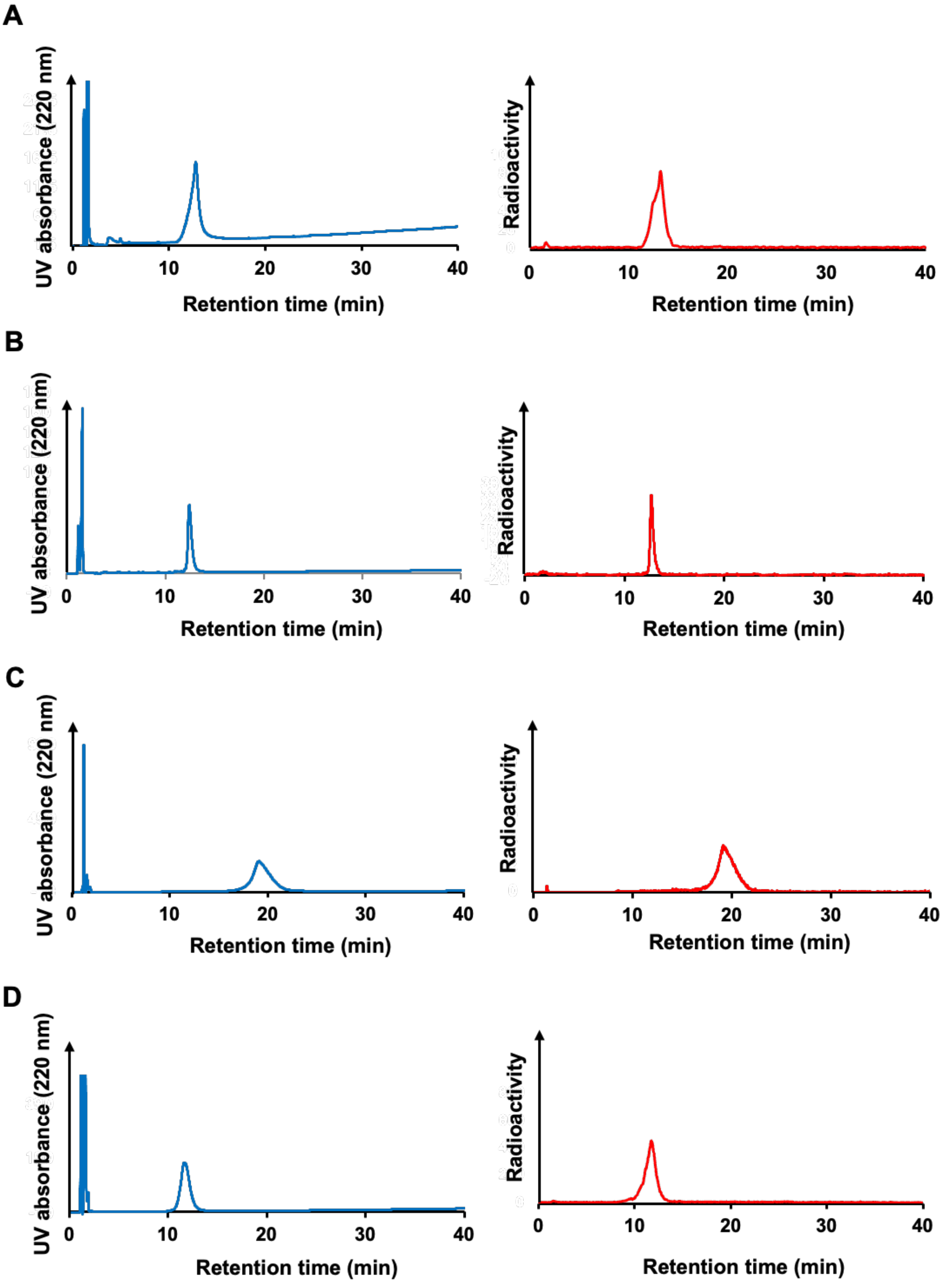

Figure S3. HPLC chromatograms of purified [^{67}Ga]Ga-NOTA-LCP1 (A), [^{67}Ga]Ga-NOTA-LCP2 (B), [^{67}Ga]Ga-NOTA-LCP3 (C), and [^{67}Ga]Ga-NOTA-LCP4 (D), analyzed by co-injection with the corresponding non-radioactive Ga-NOTA peptides. The analyses were performed using gradient elution of 0.1% TFA in H_2O and 0.1% TFA in CH_3CN for 40 min. The gradient conditions were 85/15–70/30 (A), 85/15–70/30 (B), 86/14–82/18 (C), 86/14–75/25 (D), with a flow rate of 1.5 mL/min.

s

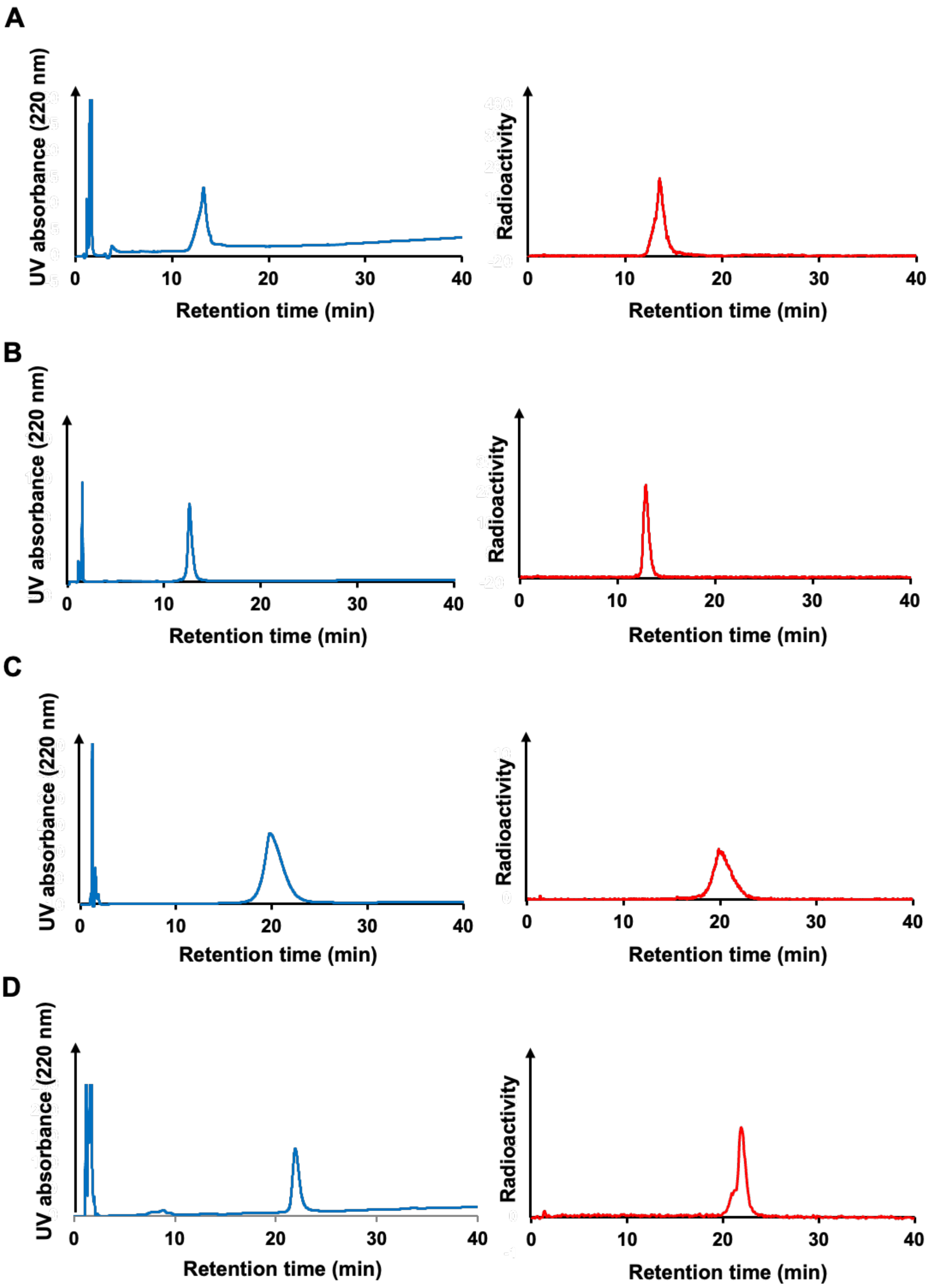

Figure S4. HPLC chromatograms of purified [^{67}Ga]Ga-NOTA-NCP1 (A), [^{67}Ga]Ga-NOTA-NCP2 (B), [^{67}Ga]Ga-NOTA-NCP3 (C), and [^{67}Ga]Ga-NOTA-NCP4 (D) analyzed by co-injection with the corresponding non-radioactive Ga-NOTA peptides. The analyses were performed using gradient elution of 0.1% TFA in H_2O and 0.1% TFA in CH_3CN for 40 min. The gradient conditions were 85/15–70/30 (A), 85/15–70/30 (B), 86/14–82/18 (C), and 90/10–75/25 (D), respectively, with a flow rate of 1.5 mL/min.

Table S2. Biodistribution of radioactivity of [^{67}Ga]Ga-NOTA peptides 1 h after intravenous administration in HCT116 tumor bearing mice.

Tissues	Biodistribution (% ID/g)			
	[^{67}Ga]Ga-NOTA peptides			
	[^{67}Ga]Ga-NOTA-LCP1	[^{67}Ga]Ga-NOTA-NCP1	[^{67}Ga]Ga-NOTA-LCP3	[^{67}Ga]Ga-NOTA-NCP3
Blood	0.28 (0.21)	0.45 (0.14)	0.46 (0.11)	0.41 (0.17)
Liver	19.08 (4.09)	17.42 (4.39)	17.19 (10.81)	20.82 (8.51)
Kidney	129.19 (23.14)	121.85 (30.91)	96.60 (38.43)	73.34 (28.63)
Intestine	0.44 (0.18)	0.53 (0.26)	0.63 (0.19)	0.54 (0.15)
Spleen	3.94 (0.92)	3.15 (1.08)	4.37 (1.39)	5.38 (0.74)
Lung	0.93 (0.27)	1.08 (0.32)	1.58 (0.43)	1.36 (0.46)
Stomach‡	0.18 (0.14)	0.22 (0.05)	0.19 (0.10)	0.12 (0.03)
Pancreas	0.31 (0.17)	0.64 (0.75)	0.38 (0.13)	0.46 (0.40)
Heart	0.53 (0.28)	0.43 (0.22)	0.44 (0.14)	0.56 (0.21)
Brain	0.03 (0.01)	0.04 (0.05)	0.04 (0.02)	0.04 (0.02)
Muscle	0.54 (0.14)	0.96 (0.23)	0.40 (0.11)	0.43 (0.22)
Tumor	1.83 (0.32)	1.80 (0.51)	0.97 (0.25)	0.95 (0.24)
	Tumor-to-nontumor ratios			
Tumor/Blood	8.29 (3.41)	4.39 (2.07)	2.20 (0.69)	2.54 (0.78)
Tumor/Muscle	3.54 (0.92)	1.91 (0.51)	2.47 (0.52)	2.70 (1.32)

Expressed as % injected dose per gram. Each value represents the mean (SD) for five animals.

‡ Expressed as %ID.
